# Endometrioma of the Liver: A Case Report and Review of the Literature

**DOI:** 10.1155/2019/4734606

**Published:** 2019-06-04

**Authors:** Prachi Rana, Shida Haghighat, Hyosun Han

**Affiliations:** ^1^Department of Internal Medicine, Los Angeles County Hospital/University of Southern California, USA; ^2^Department of Gastroenterology and Liver Diseases, Los Angeles County Hospital/University of Southern California, USA

## Abstract

Hepatic endometriosis is a rare form of endometriosis first described by Finkel in 1986. A thorough review of the literature revealed 28 cases of hepatic endometriosis. This unusual condition offers several diagnostic challenges due to its variable appearance on imaging and need for histologic analysis to establish a definitive diagnosis. We present a 42-year-old female initially treated for presumed hydatid cyst that was later found to be endometriosis in the liver. The case highlights the importance of considering endometriosis in the differential for a patient presenting with a solitary liver mass regardless of age and previous history of endometriosis.

## 1. Introduction

Endometriosis is characterized by the presence of endometrial tissue outside of the uterine cavity. It is a benign condition most commonly noted in the uterus, fallopian tubes, ovaries, and local pelvic peritoneum. Atypical endometriosis, when the condition is found in extrapelvic regions, is rare [1]. While uncommon, atypical endometriosis has been described in remote sites including the GI tract, diaphragm, skin, lung, pleura, kidney, and pancreas. The only organ in the abdomen that is refractory to endometriosis is the spleen [2].

Hepatic endometriosis is one of the most rare forms of extrapelvic endometriosis, first described in 1986 [3]. Only 28 cases have been reported in the English literature. We herein present the 29th case of hepatic endometriosis, a 42-year-old female initially treated for presumed hydatid cyst that was later found to be endometriosis in the liver. This rare condition offers several diagnostic challenges. We offer an exhaustive review of the literature focusing on advances in the clinical manifestation, patient characteristics, pathogenesis, and diagnostic workup of the condition.

## 2. Case Presentation

A 42-year-old multiparous woman presented with episodic, severe right upper quadrant pain associated with nausea and vomiting. Her past surgical history included a hysterectomy and left oophorectomy for unclear reasons. Several months prior she presented to another hospital for similar symptoms and was diagnosed with a hepatic mass. Physical examination demonstrated right upper quadrant tenderness without any palpable masses. Liver function and viral serologies for hepatitis B and C were normal. Tumor markers demonstrated normal CA 19-9 and AFP, with mildly elevated CA-125 40 U/mL (normal <38U/mL).

**Figure 1:**
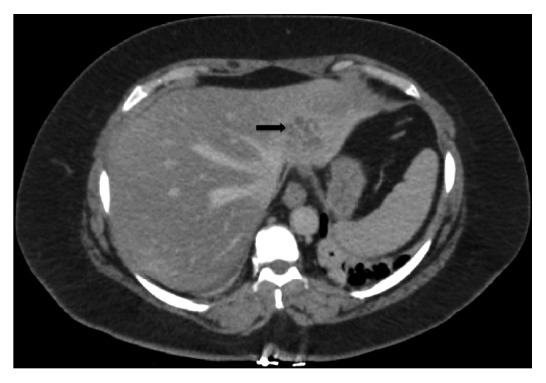
Computed tomography showing 3.2 x 4.0 x 1.8 cm multiseptated cystic lesion in the left hepatic lobe.

Computed tomography with intravenous contrast showed a 3.2cm x 4cm x 1.8cm multiseptated cystic lesion in the left hepatic lobe and an ill-defined heterogeneous hyperdensity within the peripheral right hepatic lobe measuring 3cm x 1.3cm (Figure 1). Ultrasound-guided fine needle aspiration and core biopsy of the left hepatic lesion were inconclusive.

**Figure 2:**
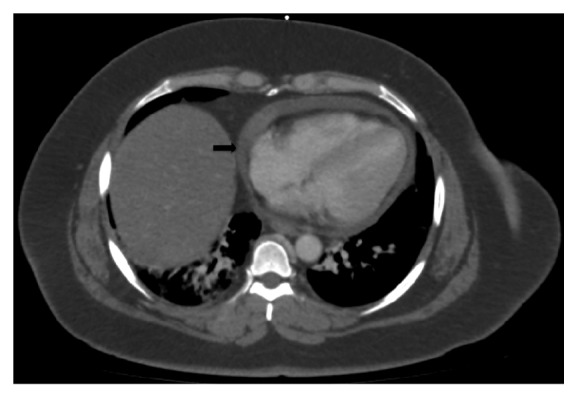
Computed tomography showing small pericardial effusion.

**Figure 3:**
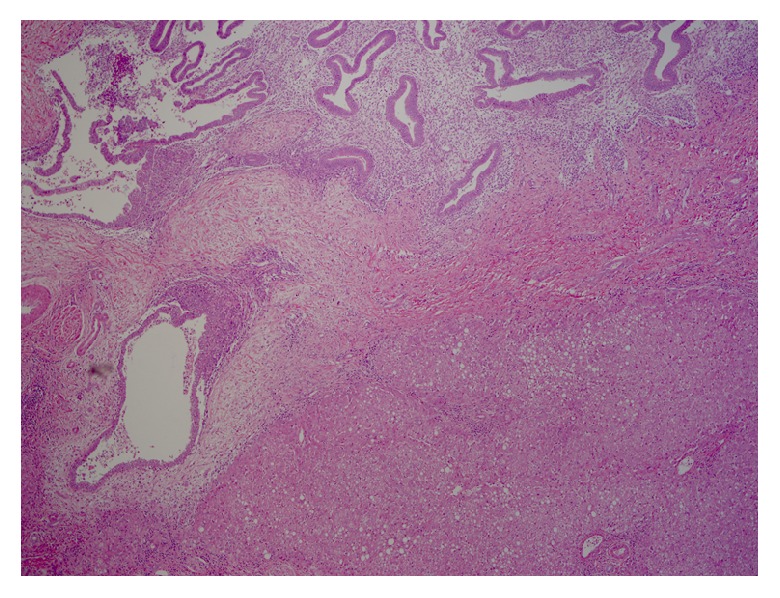
Low-power view of the interface between an endometriotic nodule and liver parenchyma. Notice the large endometrial type glands surrounded by endometrial type stroma in the superior portion of the image. These are separated from the liver parenchyma (lower right portion) by a band of fibrosis.* The liver parenchyma displays macrovesicular steatosis.* Hematoxylin & Eosin, 40x.

**Figure 4:**
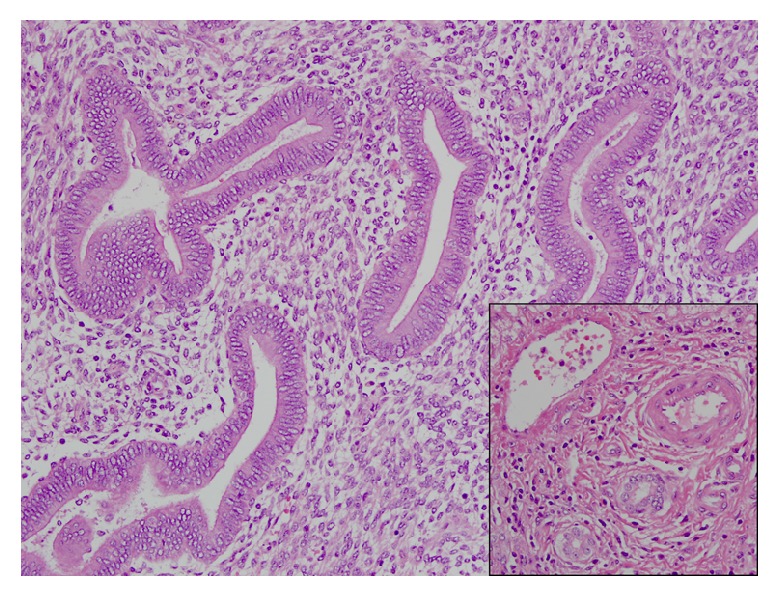
High-power view of an endometriotic nodule. Endometrioid type glands of varying shape and size show characteristic columnar lining, within a cellular endometrial type stroma.* The inset shows a portal tract for comparison (from top to bottom: vein, artery, bile ducts); notice the small round shape of the bile ducts and lack of cellular stroma.* Hematoxylin & Eosin, 200x.

Further workup revealed a positive Echinococcal IgG antibody and she was started on Albendazole for a presumed hydatid cyst. After completion of therapy, she was scheduled for complete left lateral hepatic resection. However, she presented again several weeks later with progressive right upper quadrant pain. At this time repeat computed tomography redemonstrated the left hepatic mass which was unchanged in size but did not show the right hepatic lesion. Imaging also revealed a new pericardial effusion that was not present on previous imaging (Figure 2). Her liver tests were the following: AST 485 U/L (normal 10-40 U/L), ALT 308 U/L (normal 5-40 U/L), ALP 50 U/L (normal 35-104 U/L), and total bilirubin 0.5 mg/dL (normal <1.0 mg/dL). Given the concern for pericardial involvement, she urgently underwent a laparoscopic left partial hepatectomy (segment II and partial segment III). The postoperative course was uneventful. Final pathology was consistent with hepatic endometriosis (Figures 3 and 4). After 2 months of follow-up, the patient was asymptomatic and liver tests normalized. She was started on medroxyprogesterone acetate and remains well to date.

## 3. Discussion

Endometriosis is a common gynecologic disease characterized by the presence of endometrial glands and stroma outside of the uterus. It affects 5-15% of women of reproductive age. Pelvic endometriosis involves the ovaries, fallopian tubes, uterine ligaments, Pouch of Douglas, and surrounding peritoneum [4]. A more rare form of endometriosis, extrapelvic, includes involvement of gastrointestinal tract, urinary system, thoracic cavity, kidneys, and pancreas. The exact prevalence of extrapelvic endometriosis is unknown but is thought to present in an older population with a median age of 34-40 years [4].

There is no clear consensus or unifying theory of the exact pathophysiology of extrapelvic endometriosis. Several theories have been proposed; however no theory alone accounts for the development of extrapelvic endometriosis, suggesting a multifactorial nature to the disease.

The classic theory of retrograde menstruation proposes that reflux of endometrial fragments through the fallopian tubes during menstruation results in implantation of the peritoneal cavity. Though retrograde menstruation is a common phenomenon seen in up to 90% of healthy women, not all of these women develop extrapelvic endometriosis [5].

The coelomic metaplasia theory suggests that endometriosis develops from metaplasia of the peritoneal epithelium possibly due to environmental or genetic factors [5, 6]. The induction theory suggests that defects in embryogenesis give rise to endometrial like tissue. The Müllerian ducts give rise to the female genitourinary tract. In males, this structure dissolves under the influence of anti-Müllerian hormone. However remnants of this structure may persist and differentiate later in life into endometriotic like tissue due to the presence of excesses in endogenous or exogenous estrogen as is seen in men with chronic liver disease and prostate cancer [5, 7].

While these theories account for endometriosis within the peritoneal and pelvic cavity and provide some insight into its pathogenesis in men, they do not account for the cases of disseminated endometriosis seen in cases of lymph nodes, thoracic cavity, and liver involvement, as seen in our patient. Whether these original cells originate in the uterus or the peritoneal cavity, the theory that endometriotic tissue disseminates through lymphatic spread offers a plausible explanation for the manifestation of hepatic endometriosis [5].

A thorough review of the literature revealed 28 cases of hepatic endometriosis. Our report adds one case to this rare clinical finding; herein we present the twenty-ninth case of hepatic endometriosis. Tables 1 and 2 summarize the previously reported cases and ours, comparing the presentation, imaging, treatment, and pathologic features. In this review, the patient age ranged from 21 to 62 years, with a mean of 41.5 years. Of the 19 cases that reported parity, ten were nulligravid and nine were either uni- or multiparous, thus demonstrating that pregnancy and childbirth have no bearing on hepatic endometriosis. Six of 29 (21%) patients were postmenopausal, thus showing this condition is not limited to women of reproductive age and that the diagnosis should be considered in postmenopausal women. Twelve of 29 (41%) had a prior history of endometriosis; thus the diagnosis should not be limited only to patients with a known history of endometriosis. A significant portion of these patients had prior abdominopelvic surgery—at least half (51%) had prior pelvic surgery, and 41% had a hysterectomy, suggesting that endometrial tissue seeding during surgery later resulted in the development of hepatic endometriosis. The majority (90%) of patients described in the literature had epigastric or right upper quadrant pain; only two patients complained of characteristic cyclic pain related to menses. Only three patients were asymptomatic and their condition was diagnosed incidentally. In one peculiar case, however, the patient presented with flu-like symptoms and right shoulder pain, misdiagnosed as pneumonia initially [8].

Abdominal US, CT, and MRI are the imaging modalities most frequently used. Typical US findings include well-defined cystic masses with solid components and septations. The majority of CT reports show low density, heterogenous cystic lesions that are either nonenhancing or poorly enhancing. Calcifications have been reported along with irregular soft tissue components but can be variable. Finally, MRI usually demonstrates signal intensity on T1- and T2-weighted images, similar to that of normal endometrium. However, because endometrial implants can exhibit various degrees of hemorrhage due to hormonal stimulation, implants may demonstrate a spectrum of appearances depending on the age of the hemorrhage but can be variable [9].

Of the 19 cases that reported lab values, 79% had normal liver tests. Three cases exhibited mild transaminitis, and a fourth case, ours, had markedly elevated transaminases with AST 485 U/L and ALT 305 U/L.

Excluding two, all patients underwent surgery for treatment. The most common surgery was hepatectomy via laparotomy (59%). Other surgical techniques included ultrasonic cyst manipulation. In the two nonsurgical cases, those patients were treated with danazol alone [9, 10]. Tumor size ranged from 1 to 30cm, with mean tumor size 9.8 cm.

The final diagnosis can only be made by histopathologic analysis. The differential diagnosis includes both benign and malignant conditions, as echinococcal cyst, abscess, hematoma, cystadenoma, and malignant cystic neoplasm, such as cystadenocarcinoma or metastatic disease. Method of diagnosis was largely by histologic analysis after surgery in 90% of cases. Only four patients underwent CT-guided percutaneous biopsy prior to surgery, with only three yielding a diagnosis of endometriosis and one case, ours, yielding inconclusive results. Histopathologic examination of the tumors was consistent with endometriosis as evidenced largely by fibrous capsules with internal epithelial lining containing endometrial glands and stroma. Furthermore, although malignant transformation of endometriosis is a rare event, occurring commonly in the ovary, there were two cases of malignancy reported, one adenosarcoma and one low-grade endometrial stroma sarcoma [11, 12]. Of the eight cases that reported on immunostaining, all eight cases were positive for estrogen and progesterone receptor, consistent with endometriosis. Five cases reported on further immunohistochemistry markers that included CD10 (in endometrial tissue) and/or CK7 (in glandular tissue) [13–17].

Hepatic endometriosis is a rare form of endometriosis. This unusual condition offers several diagnostic challenges but should be considered in the differential in any female presenting with a solitary hepatic mass, regardless of age and previous history of endometriosis.

## Figures and Tables

**Table 1:** Patient characteristics, presentation, and treatment of case reports of hepatic endometriosis.

Author, Year	Age	Parity	Pre/Post-Menopausal	Prior Pelvic Surgery	Hysterectomy	History of Endometriosis	Symptoms	Method of Diagnosis	Treatment
Asran, 2010	61	Unknown	Post	Salpingo-oophorectomy	Yes	Yes	Post prandial epigastric pain	CT guided percutaneous liver biopsy	N/A
Bouras, 2013	35	Nulliparous	Pre	No	No	No	Recurrent, intermittent epigastric pain	Surgery	L lateral hepatic sectionectomy by laparotomy
Chung, 1998	40	Multiparous	Pre	L ovarian cystectomy	No	Yes	Asymptomatic	Surgery	Cyst enucleation
De Riggi, 2016	27	Nulliparous	Pre	No	No	No	Painless abdominal mass	Surgery	L hepatectomy by laparotomy
Finkel, 1986	21	Uniparous	Pre	L fallopian tube cyst removal	No	No	Episodic sharp, epigastric pain associated with nausea and vomiting not related to menses	Surgery	Cyst enucleation + Danazol
Fluegen, 2013	32	Nulliparous	Pre	No	No	No	RUQ pain	Surgery	Ultrasonic pericystectomy
Goldsmith, 2009	48	Nulliparous	N/A	Salpingo-oophorectomy	Yes	Yes	Relapsing/remitting chronic RUQ pain	Surgery	Nonanatomic resection, laparotomy, ultrasonic cyst aspiration
Groves, 2003	52	N/A	N/A	Oophorectomy	Yes	No	RUQ pain	Surgery	R hepatectomy
Hertel, 2012	44	N/A	N/A	Oophorectomy	Partial	no	Sudden onset upper abdominal pain	Surgery	Partial hepatectomy
Huang, 2002	56	N/A	Post	Salpingo-oophorectomy	Yes	Yes	Intermittent epigastric pain not associated with menses	Surgery	L hepatic lobectomy by laparotomy
Inal, 2000	25	N/A	Pre	No	No	Yes	Pelvic pain, mass and rectal hemorrhage	Percutaneous CT guided biopsy	Danazol
Jelovsek, 2004	52	Uniparous	Post	Salpingo-oophorectomy	Yes	Yes	Flu like symptoms, pleuritic chest pain	Surgery	Leuprolide, then resection via laparotomy
Keramidaris, 2018	40	Multiparous	Pre	No	No	No	Asymptomatic, incidental	Surgery	Cystectomy by laparotomy
Khan, 2002	31	N/A	N/A	Hysterectomy, salpingo-oophorectomy	Yes	Yes	Malaise, abdominal distention	Surgery	R hepatectomy + goserelin
Khan, 2002	61	N/A	Post	No	No	Yes	RUQ pain	Surgery	R hepatectomy
Liu, 2015	36	Uniparous	Pre	No	No	No	RUQ pain prior to menstruation	Surgery	Pericystectomy
N'Senda, 2002	54	Uniparous	Post	Hysterectomy, oophorectomy	Yes	No	RUQ pain for 1 year	Surgery	Right hepatectomy by thoracolaparotomy
Nezhat, 2005 (1)	36	Nulliparous	N/A	No	No	No	Cyclic epigastric pain for 1 year	Surgery	Cyst removal by CO2 laser laparoscopically
Nezhat, 2005 (2)	30	Nulliparous	N/A	No	No	Yes	Chronic pelvic pain, dysmenorrhea, and painful bowel movements	Surgery	Laparoscopic removal of liver mass
Reid, 2003	46	Nulliparous	N/A	Oophorectomy	Yes	yes	RUQ pain	Surgery	R hepatectomy + goserelin
Rivkine, 2013	51	Multiparous	N/A	Hysterectomy	Yes	no	Epigastric pain, vomiting	Surgery	L lobectomy by laparotomy
Roesch-Dietlan, 2011	25	Nulliparous	Pre	No	No	No	Relapsing/remitting RUQ pain	Surgery	Incidentally found during laparoscopic cholecystectomy, treated with danazol
Rovati, 1990	37	Nulliparous	Pre	No	No	Yes	Chronic, acyclic epigastric pain	Surgery	Segmentectomy by laparotomy + Danazol
Schuld, 2011	39	Uniparous	Pre	No	No	No	RUQ pain, cough	Surgery	Segmentectomy, transdiaphragmatic pulmonary wedge resection
Sherif, 2016	44	N/A	Pre	Hysterectomy	Yes	Yes	RUQ pain and vomiting	CT guided core biopsy	Segmentectomy
Tuech, 2003	42	Nulliparous	N/A	No	No	No	Chronic, acyclic epigastric pain	Surgery	Deroofing & cystectomy
Verbeke, 1996 (1)	34	N/A	Pre	No	No	No	Acute abdomen	Surgery	R hemihepatectomy
Verbeke, 1996 (2)	62	N/A	Post	Yes	No	No	RUQ pain	Surgery	Cholecystectomy, L hepatectomy
Rana, 2019	42	Multiparous	Pre	Hysterectomy, L oophorectomy	Yes	No	Severe RUQ pain, N/V	Surgery	L partial hepatectomy

**Table 2:** Imaging features of case reports of hepatic endometriosis.

Author, Year	US	CT	MRI
Asran, 2010	N/A	Multiple, irregularly shaped, heterogeneous, low density lesions scattered throughout the liver	N/A
Bouras, 2013	N/A	10cm cystic lesion with a fatty component and calcifications	10cm cystic lesion with a fatty component and calcifications
Chung, 1998	6.4 cm x 3 cm x 2.5 cm septated cyst	Low density hepatic cyst, with undulating wall but no obvious septations	N/A
De Riggi, 2016	N/A	30cm hepatic cyts in the L love reaching segments IV, V, VIII	
Finkel, 1986	12.5 x12x9.5 cm cystic mass in L lobe with possible septations	12cm smooth-walled cystic lesion without septations	N/A
Fluegen, 2013	N/A	N/A	9.5cm x 12cm lobulated cyst in segments IV, V, VIII
Goldsmith, 2009	9 x 11cm cystic mass in segment IV. The wall appeared thick with complex septae.	N/A	11 x13 cm cystic mass in segments IV and VIII with incomplete septations
Groves, 2003	Bilateral lesions, largest in R posterior lobe 12 x 9cm	N/A	N/A
Hertel, 2012	N/A	N/A	9.5x9.1x11.2cm cystic mass with a thickened wall in R hepatic lobe
Huang, 2002	N/A	9x6cm well circumscribed cystic mass with irregular soft tissue components	N/A
Inal, 2000	Round, well defined and heterogeneous including anechoic cystic and echogenic solid components with septations and solid components	Round, well circumscribed heterogeneous mass with septations. Fine punctate/nodular calcifications at the periphery of the lesion	A lobulated but well-demarcated subcapsular mass in the posterior segment of R lobe of the liver
Jelovsek, 2004	N/A	11 x7cm mass	N/A
Keramidaris, 2018	Large cystic lesion between L and R lobe of the liver	none	Multiseptated cystic lesion 10.3x7.8x7.7 cm in the L lobe, segments IV, II, III
Khan, 2002	Large mass in R lobe and small in L lobe	large non-enhancing lobulated mass in R lobe and mass in L lobe; portal vein thrombosis	N/A
Khan, 2002	N/A	Large mass occupying the entire R lobe	N/A
Liu, 2015	6 cm lesion in L lobe (segment III)	6.5 x6cm loculated cystic lesion in segment III, wall with thick complex septae	N/A
N'Senda, 2002	N/A	Huge heterogeneous hypodense mass partially enhanced after contrast injection; cystic changes w/ fluid levels	Heterogeneous mass on both T1-, T2- and T1-weighted image after gadolinium injection; cystic changes w/ fluid levels
Nezhat, 2005 (1)	3-cm hepatic cyst in the far caudal aspect of the right lobe of the liver	3-cm hepatic cyst in the far caudal aspect of the right lobe of the liver	N/A
Nezhat, 2005 (2)	Normal findings	N/A	Normal findings
Reid, 2003	10cm mass with echogenic margins and internal debris	Low density lesion	N/A
Rivkine, 2013	80 x 75 mm intraparenchymal hepatic necrotic tumor	Hypovascularized, cystic mass in the L liver lobe with hemorrhagic contents, no septations	Cystic mass in segments II and III
Roesch-Dietlan, 2011	No masses, multiple small gallstones	N/A	N/A
Rovati, 1990	10cm cystic mass with septations	Multilocular 10cm cyst with fine calcifications in the wall	N/A
Schuld, 2011	N/A	N/A	6.8 x2.3 cm in diameter in the right basal lung and peripheral bile ducts
Sherif, 2016	3cm complex cyst in R lobes	3cm well defined hypodense subcapsular lesion in R lobe with heterogeneous peripheral enhancement in the venous and delayed phases	Subcapsular partially cystic focal lesion with possible hemorrhagic content and heterogeneous peripheral enhancement
Tuech, 2003	N/A	24cm smooth walled cystic lesion without septations in the R lobe	N/A
Verbeke, 1996 (1)	N/A	Cystic tumor in R lobe of liver, with reactive enlargement of L hepatic lobe	Cystic tumor in R lobe of liver, with reactive enlargement of L hepatic lobe
Verbeke, 1996 (2)	Cyst (12 x 10 x 7.5 cm) in the left liver lobe, located near the gallbladder and the liver hilus, which partially compressed the proximal ductus choledochus.	Cyst (12 x 10 x 7.5 cm) in the left liver lobe, located near the gallbladder and the liver hilus, which partially compressed the proximal ductus choledochus.	N/A
Rana 2019	N/A	3.2cm x 4cm x 1.8cm multi-septated cystic lesion in the left hepatic lobe	N/A
